# Neuropsychological Assessment of Older Adults With Virtual Reality: Association of Age, Schooling, and General Cognitive Status

**DOI:** 10.3389/fpsyg.2018.01085

**Published:** 2018-06-29

**Authors:** Camila R. Oliveira, Brandel J. P. Lopes Filho, Cristiane S. Esteves, Tainá Rossi, Daniela S. Nunes, Margarida M. B. M. P. Lima, Tatiana Q. Irigaray, Irani I. L. Argimon

**Affiliations:** ^1^Postgraduate Program in Psychology, Faculdade Meridional IMED, Passo Fundo, Brazil; ^2^Postgraduate Program in Biomedical Gerontology, Pontifícia Universidade Católica do Rio Grande do Sul, Porto Alegre, Brazil; ^3^Faculty of Psychology, Universidade de Coimbra, Coimbra, Portugal; ^4^Postgraduate Program in Psychology, Pontifícia Universidade Católica do Rio Grande do Sul, Porto Alegre, Brazil

**Keywords:** virtual reality, cognition, executive functions, aging, schooling

## Abstract

The development of neuropsychological assessment methods using virtual reality (VR) is a valid and promising option for the detection of cognitive impairment in the older people, focusing on activities composed of tasks of multiple demands. This study verified the association of age, schooling, and general cognitive status on the performance of neurologically healthy older adults in ECO-VR, a VR task of multiple demands for neuropsychological assessment. A total of 111 older adults answered a sociodemographic questionnaire, the Mini Mental State Examination, the Vocabulary subtest of the Wechsler Intelligence Scale for Adults (third edition), and the ECO-VR. Correlation analyses, multiple linear regression, and comparisons between groups (effects by age and schooling groups) were used to evaluate the results. The ECO-VR total score was significantly associated with age, years of education, MMSE, and Vocabulary subtest. The linear regression models identified that age was the main predictor for total score and rule breaking of ECO-VR. According to the univariate analysis, it was identified the main effect of age group and schooling group in the total ECO-VR score, but there was no interaction effect. The results are discussed in order to understand the role of sociodemographic characteristics in the performance of older adults in a VR task of multiple demands. It was also verified the possibility use of VR for neuropsychological assessment of older adults.

## Introduction

Episodic memory and executive functions are among the main cognitive changes in normal aging process, directly impacting the functional capacity of older people (Salthouse, 2010; Harada et al., 2013). The deficit performance in these functions is also described in mild cognitive impairment (MCI) and in dementia, e.g., Alzheimer’s Disease – AD (Salthouse, 2014). In order to detect significant losses in these populations, several neuropsychological tests are administered, contributing to the diagnosis of neurocognitive disorders (Plancher et al., 2012).

However, there is a dissociation between the results found in the classic tests of cognitive functions and the difficulties reported by the older adults and their relatives (Mendonça et al., 2016), in other words, the tests do not detect the occurrence of cognitive deficits while the older adults and their relatives affirm that there are damages. Generally, the investigations are made through self-report questionnaires, which are prone to subjective judgment of informer. In this sense, the development of ecological tasks has received prominence in the field of clinical neuropsychology, meeting the search for more effective alternatives in the investigation of functional characteristics. In this context, the development of neuropsychological assessment methods using virtual reality (VR) is a valid and promising option for the detection of cognitive impairment in the elderly (Okahashi et al., 2013), focusing on activities composed of tasks of multiple demands.

The researcher or clinician, when using the VR in the assessment of cognitive functions, will have greater control over the presentation of stimuli and the modulation of difficulty tasks levels. It is also possible to construct interactive environments more appropriate with the reality of the elderly adult (Plancher et al., 2012); in other words, theoretically with more expressive ecological validity. Classical neuropsychological tests, usually elaborated from experimental and laboratory perspectives, measure cognitive domains in isolation. The VR technique provides a dynamic and complex field of performance of these functions during the performance of activities that simulate those performed in the real world (Allain et al., 2014).

Until the present moment, most studies that aim to investigate through VR tasks the cognitive changes associated with aging, focused on clinical populations and assessment of navigational skills (Carelli et al., 2011; Plancher et al., 2012; Yamamoto and Degirolamo, 2012; Allain et al., 2014; Cipresso et al., 2014; Tarnanas et al., 2014), which comprise the ability of individuals to be guided by different environments. Although research with clinical groups are required to demonstrate the ability of these tasks to identify diagnostic frames, such as MCI and AD, other variables that contribute to cognitive performance, age and schooling, should also be considered.

Clinical neuropsychology searches to dissociate the cognitive alterations attributed to neurodegenerative diseases and those related to old age or influenced by other sociodemographic characteristics. In the normal aging process, there is a reduction in processing speed, which directly impacts the performance of mnemonic and attentional skills, but not necessarily the accuracy of responses (Salthouse, 2013). These variations would be consequences of structural and functional brain modifications, advancement of age characteristic, and not necessarily attributed to the manifestation of pathologies (Cabeza, 2002; Davis et al., 2008).

On the other hand, the years of formal education contribute to the construction and diversification of synaptic networks, attributing better capacity of information association and (re)organization of the brain in cases of neurological injuries (Steffener et al., 2014). Thus, schooling is a significant variable of predictive power to occurrence of cognitive deficits after brain damage and to determine the evolution of neurodegenerative diseases (Kim et al., 2014; Mortamais et al., 2014; Pinter et al., 2014; Salthouse, 2014; Howrey et al., 2015; Tifratene et al., 2015), and is one of the main variables of cognitive reserve (Habeck et al., 2017). It is emphasized that in VR studies focused on measuring the cognitive functions of healthy older adults, the analysis of the role of age and schooling is still incipient. Thus, the present article presented two objectives: (1) to verify the association of age, schooling, and general cognitive status on the performance of neurologically preserved older adults in the ECO-VR, ecological task in VR of multiple demands for neuropsychological assessment; and (2) to compare the performance of the older adults in different age groups and educational levels in the ECO-VR.

## Materials and Methods

### Participants

The sample was consisted by 111 older adults recruited for convenience and community residents. Participants aged over 60 and literate were included in the study. The exclusion criteria were: (a) score below the cutoff point in the Mini Mental State Examination – MMSE (Folstein et al., 1975, adapted by Chaves and Izquierdo, 1992), according to schooling for the population of southern Brazil (Kochhann et al., 2010): <22 for 1–5 years of study, <23 for 6-11; and <24 for 12 or more years of study; (b) score ≥ 6 in the Geriatric Depression Scale, short version – GDS-15 (Yesavage et al., 1982, adapted by Almeida and Almeida, 1999); (c) score ≥ 2 on the CAGE questionnaire (Mayfield et al., 1974, adapted by Masur and Monteiro, 1983); d) deficit and/or uncorrected vision and hearing at the time of assessment; (e) neurological diseases (such as cerebrovascular accident or traumatic brain injury) or coexisting psychiatric disorders (mood or anxiety disorders), investigated by self-report; and f) use of benzodiazepines and/or antipsychotics. The assessments were conducted individually in a single session of approximately 90 min. The written informed consent of all the participants was obtained and the Research Ethics Committee of the Pontifical Catholic University of Rio Grande do Sul approved the development of this study (CAAE 15961113.0.0000.5336).

### Instruments and Procedures

#### Sociodemographic, Clinical and Cognitive Characteristics

Participants answered the sociodemographic and clinical questionnaire with questions about age, years of study, gender and medical history. Previous experience with the computer was investigated through a dichotomous question (“yes” or “no”) and problems with alcohol use were verified with the CAGE questionnaire. The general cognitive level was evaluated by the MMSE and by the Vocabulary subtest of Wechsler Intelligence Scale for Adults, third edition – WAIS-III (Wechsler, 1997, adapted by Nascimento, 2004). The MMSE is a cognitive screening test that assesses temporal and spatial orientation, immediate memory, attention/concentration, late recall and language, while the Vocabulary subtest is a measure of crystallized intelligence.

#### Assessment With Virtual Reality: ECO-VR

**FIGURE 1:**
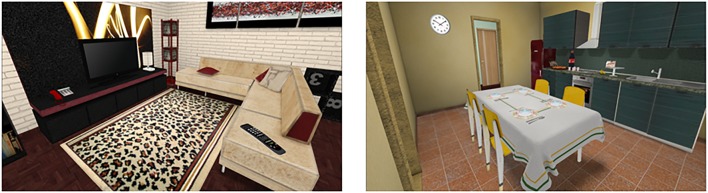
Examples of three-dimensional scenarios (room and kitchen) used in ECO-VR.

The ECO-VR (Oliveira et al., 2016) is a three-dimensional environment in VR (**Figure 1**), completely colored and textured that simulates a house. In addition to visual stimuli, auditory stimuli are included to promote greater sense of presence. For projection, a notebook with 17-inch screen and Intel processor Duo Core (2,1 GHz, 4 GB RAM) was used and ATI Mobility Radeon HD 4650 (M96) Video Card, and the virtual scenarios and objects were modeled in the NeuroVr2 software (Riva et al., 2007). The interaction with the virtual environment presented a non-immersive and first-person perspective, aiming to reduce the risk of adverse effects such as headache and dizziness, as well as facilitating the administration of the task in the clinical context (Flynn et al., 2003). The participants controlled their movements in the scenarios through joypad, through a training session in a virtual environment that simulated a square, without time limit, to be familiarized with the software and controls. The instructions and rules for performing ECO-VR activities were delivered to the participants in writing and read aloud.

The ECO-VR is composed of five tasks of multiple demands that involve activities of daily living: (1) to watch the news on television; (2) to check the messages on the answering machine; (3) to separate food and organize a meal; (4) to find a specific object in the room; and (5) to recall information related to the television news and the message on the answering machine. The rooms in the house included a living room, bedroom, bathroom, kitchen and hall, as well as characteristic objects and furniture. Performance analysis included the scores of the number of activities completed (between 0 and 24 points), the time in seconds for conclusion, rules breaking (frequency of pre-established rule violations, with scores between 0 and 21) and strategies used (frequency of use of mechanisms to assist in more effective execution of activities, such as asking for help and taking notes, with scores between 0 and 24). Except for time to conclusion and rule breaking, higher scores represent better performance. The scores construction of ECO-VR was based on the study of Raspelli et al. (2012).

### Data Analysis

The data were analyzed in the statistical package SPSS, version 22, for Windows. Descriptive statistics (mean, standard deviation, and percentage) were used in the sociodemographic characterization, and cognitive instrument scores and depressive symptoms of the participants were used to refer to ECO-VR variables. Data distribution was examined using the Kolmogorov–Smirnov test and Pearson’s correlation was used to verify associations between ECO-VR scores, age, years of study, and general cognitive status (MMSE and Vocabulary subtest). In order to investigate the predictive variables for performance in ECO-VR, multiple linear regression analysis with stepwise method was used. The predictive variables were age, schooling, total MMSE score, Vocabulary subtest score and previous experience with computer (“no” = 0 and “yes” = 1). The presence of residues and collinearity was verified by the Durbin–Watson test. Univariate analyzes were performed to verify differences in performance in ECO-VR according to age groups (60–69 and 70–85 years old) and educational levels (≤12 and ≥13 years of formal education), as well as interaction effect. It is emphasized that groups composed of educational levels were defined based on the Brazilian classification of primary and higher education. The one-way ANOVA and the chi-square test were used to verify differences in sociodemographic and clinical data, cognitive ability and ECO-VR scores between groups by age and schooling. Effect size was calculated from Cohen’s *d* and ≤0.20 were classified as an effect of low magnitude, ≤0.50 mean and ≥0.80 large (Cohen, 1988). The significant level was set at *p* < 0.05.

## Results

The sociodemographic, clinical and cognitive characterization of the sample are found in **Table 1**. Regarding the gender distribution, 17.12% (*n* = 19) were men and 82.88% (*n* = 92) were women. In addition, 33.33% (*n* = 37) reported experience with computer/notebook use, while 66.67% (*n* = 64) do not.

**Table 1:** Sociodemographic, clinical and cognitive characteristics of participants.

	*M*	*SD*	Range
Age (years)	68.79	6.11	60 – 85
Schooling (years)	13.04	4.62	2 – 25
MMSE (maximum 30)	27.56	2.01	23 – 30
Vocabulary – WAIS-III (maximum 66)	35.85	8.33	14 – 54
GDS-15 (maximum 15)	2.14	1.76	0 – 5

*MMSE, Mini Mental State Examination; WAIS-III, Wechsler Intelligence Scale for Adults, third edition. GDS-15, reduced version of the Geriatric Depression Scale*.

### Relations Between Age, Schooling and Performance in ECO-VR

The analyzes were performed based on mean ECO-VR scores, age, schooling, and general cognitive status (MMSE and Vocabulary subtest). According to results of Pearson’s correlation (**Table 2**), there was a significant negative and moderate association between age and total ECO-VR score, and positive and weak with ECO-VR rule breaking frequency. This result suggests that according to the advancement of age, the accuracy in ECO-VR decreases, while non-compliance with the pre-established rules increase. In relation to schooling, there was a significant positive and moderate association with a total score of ECO-VR, demonstrating that the number of years of formal education influenced the adequate execution of the virtual task activities.

**Table 2:** Pearson’s correlation between ECO-VR scores, age, schooling and cognitive status.

	ECO-VR			
	Total	Rule breaking	Strategies	Time
Age (years)	-0.526***	0.275**	-0.075	0.128
Schooling (years)	0.363***	0.005	0.073	-0.091
MMSE				
Time orientation	0.095	-0.115	0.012	-0.001
Space orientation	0.290**	0.068	-0.077	-0.008
Immediate Memory	0.193*	0.032	-0.007	0.084
Attention/Concentration	0.036	-0.152	-0.065	-0.026
Delayed recall	0.239*	-0.179	-0.065	-0.166
Language	0.422***	-0.024	0.024	0.030
Total	0.276**	-0.216*	-0.078	-0.082
Vocabulary – WAIS-III	0.250**	-0.228**	0.224**	0.033

*MMSE, Mini Mental State Examination; WAIS-III, Wechsler Intelligence Scale for Adults, third edition; ^∗^*p* < 0.05, ^∗∗^*p* ≤ 0.01, ^∗∗∗^*p* ≤ 0.001*.

### Relations Between General Cognitive Status and Performance in ECO-VR

Significant associations were found between ECO-VR and MMSE scores (**Table 2**). Specifically, spatial orientation, immediate memory, and late recall subtests demonstrated significant and positive correlations. Both presented low magnitude, with the total score of ECO-VR, while the language had a significant positive and moderate association. Analyzing the MMSE overall score, it was found weak and positive significant relationship with the total ECO-VR score, and negative with rule breaking. Therefore, better results in spatial orientation, verbal memory and language, as well as general cognitive ability, seem to be associated with performance in ECO-VR. Finally, in **Table 2**, the Vocabulary subtest demonstrated significant weak and positive associations with the total score and strategies, and negative with the ECO-VR rule breaking score. Thus, higher scores on this subtest also suggest better results in ECO-VR performance, as well as more frequent use of appropriate strategies and greater compliance with pre-established rules.

### Influence of Age, Schooling, and General Cognitive Status in Performance on ECO-VR

In order to identify whether performance in ECO-VR could be explained by age, schooling, general cognitive status or previous computer experience, multiple linear regression analyzes were performed (**Table 3**). In the model with the ECO-VR total score as the dependent variable, there was a strong association (*R* = 0.605; *p* ≤ 0.001) with the predictive variables. Age, years of study and Vocabulary represented 34.80% of the variance in the accuracy of ECO-VR. Standardized regression coefficients demonstrated that age was the strongest predictor. Considering the rule breaking score as a dependent variable, there was a moderate association (*R* = 0.359; *p* ≤ 0.001) with the predictive variables. Age and Vocabulary contributed with 11.20% of the variance and, again, age was the most important predictor. The final model included the scoring strategies as dependent variable. There was weak association (*R* = 0.224; *p* ≤ 0.018) between the number of strategies used to solve ECO-VR and Vocabulary tasks, which explained 4.20% of variance. Finally, considering the execution time as dependent variable, no significant explanatory models were found. It is emphasized that the experience with the computer/notebook was not a relevant predictor for any of the models investigated.

**Table 3:** Linear regression models for total scores, ECO-VR Rule breaking and Strategies

Models	*b ± SE*	*β*	*T*	CI of 95%	*R^2^*	*R^2^a*	*F*	*p*
Total								
Constant	34.149 ± 4.304		7.935	25.617 – 42.681				
Age (years)	-0.349 ± 0.057	-0.483	-6.126	-0.462 – -0.236	0.366	0.348	20.547	≤0.001
Vocabulary – WAIS-III (score)	0.122 ± 0.048	0.210	2.564	0.028 – 0.216				0.012
Schooling (years)	0.182 ± 0.079	0.191	2.289	0.024 – 0.339				0.024
Rule breaking								
Constant	-0.998 ± 3.308		-0.302	-7.556 – 5.560				
Schooling (years)	0.138 ± 0.045	0.277	3.082	0.049 – 0.227	0.129	0.112	7.971	0.003
Vocabulary – WAIS-III (score)	-0.097 ± 0.036	-0.241	-2.676	-0.168 – -0.025				0.009
Strategies								
Constant	4.901 ± 1.231		3.980	2.460 – 7.341				
Vocabulary – WAIS-III (score)	0.080 ± 0.033	0.224	2.403	0.014 – 0.146	0.050	0.042	5.773	0.018

*CI, Confidence Interval; *R^*2*^*a, *R^*2*^* adjusted.*

### Differences Between Groups According to Age and Schooling in ECO-VR

**Table 4** presents the performance comparison between age and schooling groups. In relation to age groups, both demonstrated similar cognitive performance and did not differ in gender distribution (*χ*^2^ = 0.200; *p* = 0.655) and computer/notebook experience (*χ*^2^ = 1.188; *p* = 0.276). The younger group presented more accuracy in the ECHO-VR compared to the older group, less frequent failing in pre-established rules, differences of large and medium magnitude, respectively. The analyzes, according to the groups by educational levels, in relation to cognitive performance the group with more years of study obtained a significantly superior performance in the Vocabulary subtest. There were no differences in relation to age, MMSE, gender distribution (*χ*^2^ = 0.293, *p* = 0.589) and computer/notebook experience (*χ*^2^ = 1,805, *p* = 0.179). Higher educated participants correctly completed more ECO-VR tasks than the less educated, and this result was of medium magnitude.

**Table 4:** Comparison of performance in ECO-VR scores between age groups and levels of schooling.

	Groups by Age						Groups by Schooling				
	60–69 (*n* = 65)	70–85 (*n* = 46)	*F*	*p*	*d*		≤12 (*n* = 53)	≥13 (*n* = 58)	*F*	*p*	*d*
Age (years)	64.54 ± 2.76	74.80 ± 4.16	6.840	≤0.001	–		70.25 ± 6.64	67.47 ± 5.30	2.731	0.016	–
Schooling (years)	13.37 ± 4.28	12.57 ± 5.08	3.161	0.369	–		9.13 ± 2.76	16.60 ± 2.68	0.015	≤0.001	–
MMSE	27.66 ± 1.81	27.41 ± 2.27	3.707	0.523	–		27.19 ± 2.18	27.90 ± 1.78	3.271	0.063	–
Vocabulary	35.88 ± 7.65	36.50 ± 8.24	0.427	0.696	–		34.04 ± 7.42	38.13 ± 7.88	0.106	0.008	–
ECO-VR											
Total	18.44 ± 3.66	14.84 ± 4.91	3.224	≤0.001	-0.830		15.72 ± 5.06	18.02 ± 3.81	5.328	0.011	–0.510
Rule breaking	4.37 ± 2.95	5.91 ± 3.27	0.601	0.014	-0.500		5.42 ± 3.00	4.66 ± 3.30	0.807	0.225	–0.240
Strategies	8.02 ± 2.37	7.50 ± 3.33	5.821	0.383	-0.180		7.48 ± 2.65	8.09 ± 2.96	0.000	0.271	–0.220
Time	664.03 ± 255.24	707.09 ± 220.83	0.117	0.372	-0.180		688.97 ± 297.56	676.25 ± 174.29	3.670	0.790	–0.050

*MMSE, Mini Mental State Examination; *d*, Effect size*.

According to the univariate analysis, it was identified the main effect of age (*F*_2,109_ = 15.820; *p* ≤ 0.001; observed power = 0.976) and schooling group (*F*_2,109_ = 4.391; *p* = 0.039; observed power = 0.546) in the total ECO-VR score, but there was no interaction effect. A significant main effect of age group for the rule breaking score was also found (*F*_2,109_ = 5.496; *p* = 0.021; observed power = 0.641), but there was no main effect by educational level. In the strategy and execution time scores, no major effects of age and schooling were identified.

## Discussion

The present study investigated the influence of age, schooling, and general cognitive status on the performance of ECO-VR, an ecological task in multiple demands for neuropsychological assessment of older adults, through two objectives. In the first one, the influence of age, years of study and cognitive ability (MMSE and Vocabulary) on the performance of ECO-VR in neurologically healthy older adults was verified. From the Pearson correlation analysis, it was observed that age and MMSE presented significant associations with the total score and ECO-VR rule breaking, while schooling was only related to the total score. In contrast, the Vocabulary subtest obtained associations with all ECO-VR scores, except with run time. In this sense, explanatory models for each of the ECO-VR scores were analyzed through linear regression analysis, revealing that for the total ECO-VR score there is a greater influence of age variables, schooling and Vocabulary; in the ECO-VR rule breaking score, there was a significant participation of age and Vocabulary, while in the strategies score only Vocabulary performance demonstrated relevant contribution. The implementation time of ECO-VR did not present a significant explanatory model.

The second objective was to compare the performance of age groups (60–69 and 70–85 years old) in ECO-VR. It was verified that the older adults of the younger group obtained better results in the total score and rule breaking. In other words, both demonstrated more accuracy and failed to provide a smaller amount of predefined combinations. Finally, performance in ECO-VR was compared in groups distributed according to schooling (≤12 and ≥13 years of schooling), which differed significantly only in the total score. The higher educated older adults completed more activities adequately in the task when compared to the less educated.

The results agree with the studies of Jansen et al. (2010), Plancher et al. (2010), Carelli et al. (2011), and Yamamoto and Degirolamo (2012), which verify the effect of age and schooling in the performance of neuropsychological tasks in VR, mainly in the skills of topographical orientation and spatial learning in healthy older adults. According to multiple regression data, age performed a greater role in ECO-VR scores than schooling since cognitive differences associated with aging were more emphasized when tasks simultaneously involve storage and processing of information (Salthouse, 2010, 2013, 2014).

Reasoning ability, visuospatial skills, verbal memory, and processing speed tend to decline with age, except for vocabulary ability, which has a positive association (Salthouse, 2013). However, the neural mechanisms responsible for memory processing and executive function associated with aging are still not completely defined. The loss of gray matter would be related to the cognitive loss due to aging, and hippocampal volume reduction is responsible for memory difficulties and, in the frontal regions, executive functions (Bauer et al., 2015; Salminen et al., 2016). In addition, in tasks that require mnemonic resources, functional neuroimaging studies have identified parallel activations in the temporal and frontal regions, which suggests the participation of attentional processes and executive functions as compensatory strategies in older individuals (Vannest et al., 2015; Hedden et al., 2016).

Considered a task of multiple demands, the ECO-VR is formed by a series of activities consisting of goals and subgoals and during its execution, the cooperation of numerous cognitive domains is required (Burgess et al., 2000). For example, it is necessary to recall what must be accomplished and in which order, in order to plan the sequences of actions and to solve possible problems. Throughout these activities, there is activation of memory skills and executive functions, which present changes with advancing age and impact the functionality of older individuals (Salthouse, 2014; Martins et al., 2015).

Burgess et al. (2000) using structural equation techniques, identified the performance of a multi-demand task involved, mainly, retrospective memory, prospective memory and planning. In VR studies, Logie et al. (2011) found similar results in healthy adults as they considered a multi-demand task set in a shopping center. In our findings, although no specific instruments were used to assessment different mnemonic and executive domains, there was a significant participation of spatial orientation functions, verbal (immediate and late memory), language, general cognitive ability and crystallized intelligence for the execution of ECO-VR. Other studies with VR with tasks of multiple demands also found an association with accuracy in virtual tasks in healthy older individuals (Kang et al., 2008; Raspelli et al., 2012; Okahashi et al., 2013).

Although the results of this study identified a lower contribution of schooling performance of ECO-VR, the findings in the literature are not conclusive regarding the impact of variable on cognition (Salthouse, 2014; Farina et al., 2017). It is possible that in our results the impact of schooling was not accentuated since the activities requested in ECO-VR were representative of daily life, not necessarily depend on formal education to be successfully performed. In the study by Kang et al. (2008), which used a multiple-order VR task for neuropsychological assessment in older adults, also was not found schooling effect.

The performance in ECO-VR demonstrated dependence on general cognitive ability and crystallized intelligence, mainly regarding the rule breaking and the use of strategies. Older adults with higher scores in the MMSE and in the Vocabulary subtest were more accurate, failing in rules less frequently and using appropriate techniques and strategies. The maintenance of active cognitive functioning is also present during the aging process, and contributes to the elaboration of compensatory strategies to reduce the impact of age-related changes, especially regarding the effectiveness of executive processes (Carelli et al., 2011). It is possible that some ECO-VR activities were considered more complex than others to study participants, which would explain the more expressive demand for cognitive ability. The contribution of crystallized intelligence, related to the accumulation of information based on life experiences (Salthouse, 2014), may have occurred since the requested tasks of ECO-VR were familiar to the older adults.

This study presents innovative results regarding the influence of age, schooling, general cognitive ability and crystallized intelligence on the performance of VR task for neuropsychological assessment of older people, although it is necessary to emphasize some limitations. The first is the non-inclusion of other age groups for comparison, such as young adults, besides more stratified educational levels. It was not possible to investigate differences between gender since the number of males was extremely reduced. However, it should be noted that differences in performance in ECO-VR between age and schooling groups cannot be attributed to the previous experience with the computer. All participants received similar training regarding joypad use and, according to models of regression, this variable was not predictive for ECO-VR scores. Another important limitation was the assessment of general cognitive status based only on a cognitive screening tool and one of the main subtests for assessing verbal intelligence. Neuropsychological instruments need to be included in further studies since screening tests superficially contemplate few cognitive functions.

In general, age and schooling demonstrated influence on the adequate performance of ECO-VR, as well as on the ability to follow established rules and on the use of effective strategies. It suggests that these variables also impact on the execution of daily activities that demand the activation of multiple cognitive processes. In addition, ECO-VR scores were associated with general cognitive ability as well as with crystallized intelligence. In conclusion, ECO-VR performed as an ecological task of multiple demands in VR for measuring cognitive functions of older adults through similar activities of daily life, being able to discriminate the older adults of different age groups and educational levels.

## Author Contributions

CO designed the study, contributed to data collection and analysis, and wrote the initial draft of the manuscript. BLF contributed to interpretation of data, and assisted in the preparation of the manuscript. CE contributed to interpretation of data and assisted in the preparation of the manuscript. TR and DN assisted in the preparation of the manuscript. ML contributed to interpretation of data and assisted in the preparation of the manuscript. TI designed the study, contributed to interpretation of data and assisted in the preparation of the manuscript. IA designed the study, contributed to interpretation of data, and assisted in the preparation of the manuscript. The final version of the manuscript was approved by all authors.

## Conflict of Interest Statement

The authors declare that the research was conducted in the absence of any commercial or financial relationships that could be construed as a potential conflict of interest.
